# Heavy-chain immune repertoire sequencing enables language-model prediction of antigen-specific antibodies

**DOI:** 10.21203/rs.3.rs-9710622/v1

**Published:** 2026-06-18

**Authors:** Karen Paco, Mariana Mendivil, Zihao Zhang, Sanaz Zebardast, Christian Davila, Ryan Mooney, Peace Olatoyinbo, Tristan Yang, Sebastian Bassi, Virginia Gonzales, Eva Chen, Faisal Bin Ashraf, Isabel Condori, Jonathan Felix, Rashid Alam, Jordan Lay, Malkiat Johal, Karine Le Roch, Ilya Tolstorukov, Jeniffer Hernandez, Fernando Barroso Da Silva, Stefano Lonardi, Matthew Sazinsky, Animesh Ray

**Affiliations:** Keck Graduate Institute; Keck Graduate Institute; Keck Graduate Institute; Keck Graduate Institute; Keck Graduate Institute; Pomona College; Keck Graduate Institute; Keck Graduate Institute; Toyoko Labs; Toyoko Labs; Pomona College; University of California, Riverside; Keck Graduate Institute; Keck Graduate Institute; Keck Graduate Institute; Keck Graduate Institute; Pomona College; University of California, Riverside; Keck Graduate Institute; Keck Graduate Institute; Universidade de São Paulo; University of California, Riverside; Pomona College; Keck Graduate Institute

## Abstract

Rapidly identifying antigen-specific antibodies within complex B cell repertoires is important for therapeutic antibody discovery, vaccine development, disease surveillance, and immune condition monitoring, especially for emerging pandemics. RNA deep sequencing can rapidly provide antibody sequence candidates, but predicting their binding-specificity has remained difficult. Here we show that antigen-specific antibodies can be predicted directly from mRNA-derived heavy-chain V(D)J deep sequencing of unselected immune repertoires by parameter-efficient fine-tuning of the protein language model ESM-2. We have achieved high antigen recognition accuracies across antibodies specific for SARS-CoV-2 spike protein, influenza hemagglutinin, and HIV glycoprotein gp120 antigens, respectively. Our fine-tuned language model, Antigen Specificity Predictor, when applied to unsorted peripheral blood repertoires from immunized mice by single-cell deep sequencing, could predict specific B cell receptors at high frequency, which were then experimentally validated. A significant overlap was obtained with the predicted receptors when benchmarked on previously unseen human B cell receptor sequences identified by barcoding-enabled affinity selection. In bulk mRNA-sequences of human immune repertoires, the predicted antigen-specific B cells exhibited characteristics reminiscent of biology-aware learning. Our model’s performance cannot be explained by sequence memorization. We establish that unselected heavy chain antibody sequences alone carry sufficient signal for repertoire-scale computational antibody discovery and immune profiling, thus motivating potential extension to autoantibody identification in cancer and autoimmune diseases.

## Teaser

Language models decode antibody-antigen specificity from sequence alone, enabling large-scale immune profiling without experimental selection.

## Introduction

Upon an usual immune response, antigen-specific B cells constitute about 0.005–0.1% of the peripheral immune lymphocyte repertoire (1, 2). Identifying the sequences of these rare B cell receptors (BCRs) that exhibit high affinity binding against a specific antigen eliciting the immune response is essential for vaccine design (3, 4), monoclonal antibody discovery (5–7), tracking pathogen evolution (8, 9), and vaccination effectiveness (10).

Available experimental approaches—including fluorescence-activated cell sorting with fluorescent antigens (11–13), phage and yeast display (14, 15), and hybridoma technology (16)—successfully isolate functional antibodies but are slow, requiring weeks to months of effort. High-throughput antibody repertoire sequence discovery by deep sequencing of antibody transcripts in B or T cells can identify antibody sequences (17, 18) but does not identify antibody specificity. Recent advances in single-cell RNA deep sequencing combined with generating a physical linkage between DNA-barcoded antigens and their corresponding BCRs at the single cell level, such as Linking B cell Receptor to Antigen Specificity through Sequencing (LIBRA-seq) (19) and Barcode-Enabled Antigen Mapping (BEAM) (20, 21), enable higher-throughput identification of antigen specificity. However, these methods require careful optimization of in vitro binding conditions and are expensive to conduct. These constraints limit the scale at which immune repertoires can be interrogated for antigen specificity, particularly when analyzing archived samples, conducting large cohort studies, or mining public databases compiled from standard bulk sequencing. Computational prediction of antigen specificity from sequence alone would circumvent these experimental bottlenecks.

A recent computational method utilizing antibody gene repertoire sequence analysis to identify lineages/clusters of related and selectively amplified antibody variable chain V(D)J junction sequences has been able to accomplish antibody target identification (22), but this method ideally requires time-series repertoire sequence data and antigen-specificity is inferred from the hypothesis that antigen-specific antibody sequences undergo demonstrable and selective amplification following immunization, which might not always be practicable. Deep learning bi-directional transformer models (23) such as AntiBERTa (24), and, coupled with graph neural networks, Antiformer (25) can help predict antigen-binding specificity of antibodies, but neither classifies antigen-specific antibodies from the whole immune repertoire. Similarly, deep learning models trained on paired antigen-antibody datasets have demonstrated excellent performance (26, 27), including an image-based deep learning method (28), but typically require both heavy and light chain sequences and are constrained by the availability of labeled training data (29) and typically do not classify repertoire-scale sequence data.

Recent exciting progress in protein language models (PLMs)—large neural networks pretrained on millions of natural protein sequences through self-supervised learning (30–34)—has transformed protein engineering by emulating evolutionary patterns and imposing long and short range structural constraints without explicit functional annotations. PLMs such as ESM-2 (33, 34) generate contextualized sequence representations that, when finetuned on specific tasks, have enabled advances in protein fitness prediction (35–40), stability engineering (41–43), protein binding affinity estimation (25, 44, 45), and binding affinity/fitness improvement (46). However, whether PLMs can learn to predict antigen specificity from the heavy chain alone—enabling analysis of standard bulk immune repertoire data without requiring paired sequences or experimental selection—remains unexplored. Antibody heavy chains, particularly their complementarity-determining regions (CDRs), encode significant structural and physicochemical information for antigen specificity (47). Light chains are generally important for modulating the heavy chain’s binding affinities (48), and also can be integral to antigen-binding specificity (49).

We reasoned that supervised machine learning models trained on heavy-chain-only sequences might distinguish antigen-specific from non-specific BCRs. If realized, this would enable computational screening of entire immune repertoires, which will transform vast bulk V(D)J sequence datasets into functional predictions without the need to physically link the BCRs to antigens or to obtain paired heavy and light chain sequences.

Here we demonstrate that retraining ESM-2 on curated antigen-antibody pairs yields a classifier (Antigen Specificity Predictor, or ASPred) that accurately identifies antigen-specific BCRs from unselected repertoires across multiple viral antigens. These results suggest that heavy-chain sequences alone contain sufficient predictive signals of antigen specificity that are learnable by PLMs.

## Results

**Figure 1:**
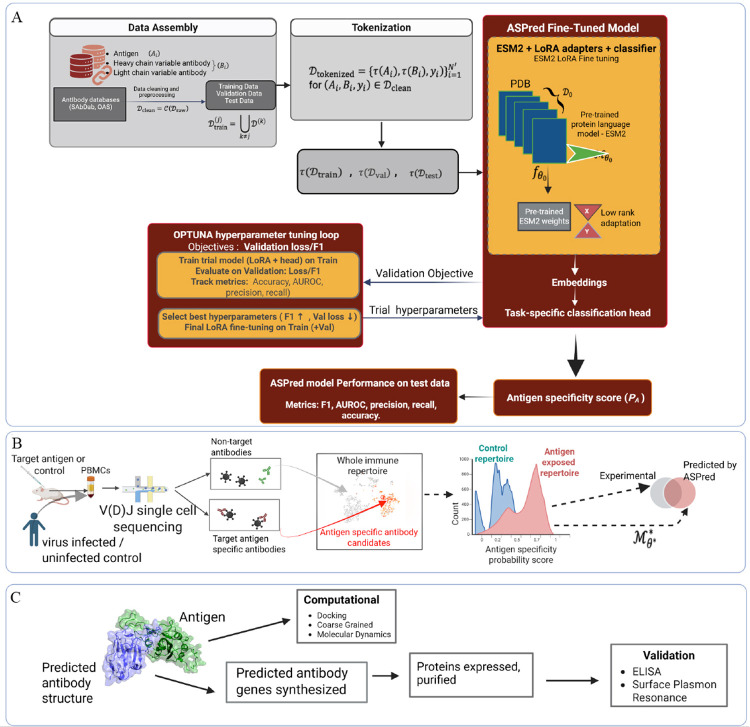
The strategy for constructing and validating ASPred. . **(A)**, Schematic of fine-tuning ESM-2 to develop ASPred. Heavy and corresponding light chain antibody sequences (with data partitions provided in the diagram) were embedded using the ESM-2 PLM, then fine-tuned via parameter-efficient LoRA adaptation. A classification head outputs the antigen specificity probability P_A_ for each antibody sequence. **(B)** Validation of ASPred predictions applied to immune repertoire data and benchmarking against experimental data. RBD-immunized mouse whole immune repertoire was obtained by V(D)J sequencing of barcoded single cells without affinity tagging with the antigen. Antigen specificity scores by ASPred were obtained, and the resulting distributions enabled prioritization of high-confidence binders over non-binders. Human V(D)J sequence data from SARS-CoV-2 infected patients with RBD specificity scores from Barcode Enabled Antigen Mapping (BEAM) were used for ASPred inference testing. The same V(D)J sequence datasets were scored and stratified by ASPred according to predicted specificity. The overlap between the experimentally observed and predicted antibodies and their frequency distributions were compared. Publicly available human SARS-CoV-2 infected whole immune repertoires (and healthy controls) sequence data were used for the inference of RBD-specificity with ASPred. **(C)** Validation of ASPred-predicted antigen specificity of antibodies in the whole immune repertoire (above) by gene synthesis, cloning, protein expression, purification, binding assays against the antigen, and molecular dynamics simulation.

Using publicly available paired antigen-antibody sequence data (Fig. 1A), we fine-tuned the general embeddings of a PLM to high predictive accuracy (ASPred), with strong performances on three different antigen classes, explored its predictions on previously unseen data, used it to make predictions to classify novel antigen-specific antibodies in mice whole-immune repertoire by single cell V(D)J sequencing and on high-throughput experimentally obtained antigen-specific human antibody sequences (Fig. 1B), validated the candidates by expression in *E. coli* as nanobodies (Fig. 1C), and examined model performance on unseen human whole immune repertoires.

### Fine-tuning ESM-2 enables accurate prediction of antigen-specific antibodies from heavy chains

**Figure 2:**
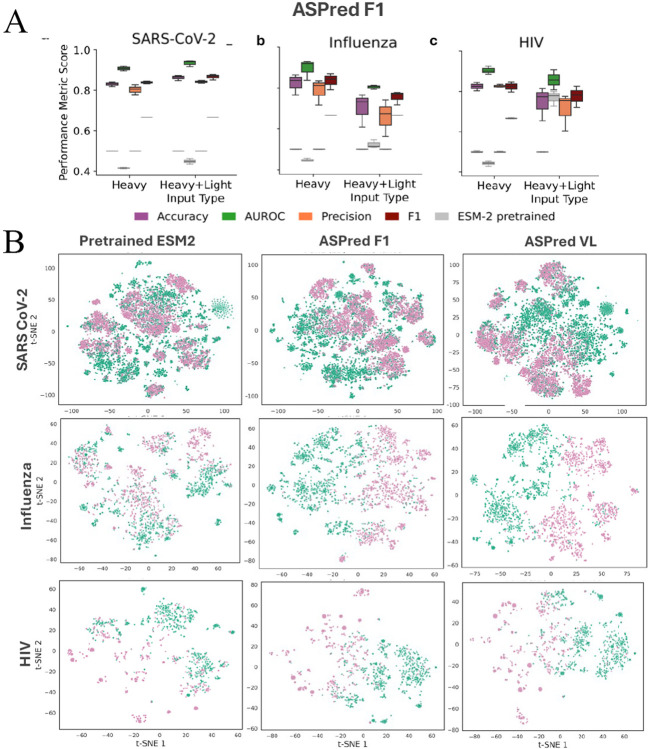
Performance evaluation of ASPred models across three antigens. Cross-validated performance of F1-optimized models in terms of accuracy, AUROC, precision, and F1, for **(A**) SARS-CoV-2 RBD (n=11,425), **(B)** influenza HA (n=2,577), and **(C)** HIV gp120 (n=1,340), using heavy, and paired heavy–light-chain inputs. The model configurations are summarized in the Supplementary Table S1. Negative examples (non-specific antibodies) were defined as antibody sequences annotated outside the target antigen class, while maintaining balanced class ratios (Supplementary Data1). **(D)** Two-dimensional t-SNE projections of sequence embeddings before fine-tuning (pretrained) and after fine-tuning (ASPredF1, ASPred-VL). Purple, BCR sequences predicted to be antigen specific; green, predicted to be not specific to the given antigen. Dataset sources and selected hyperparameters are detailed in Methods, and Supplementary Information.

To investigate whether PLMs can predict antigen specificity from heavy-chain sequences, we fine-tuned the ESM-2 transformer architecture (33) on curated datasets of antibodies with known antigen targets (Fig. 1A). We compiled training data from public repositories of CoV-AbDab (50) (Supplementary Data 1), comprising heavy-chain as well as both heavy and light chain sequences for antibodies specific to three viral antigens: SARS-CoV-2 receptor-binding domain (RBD), influenza hemagglutinin (HA), and HIV glycoprotein gp120. These choices were motivated by data availability and annotation frequencies. We first developed an initial full-parameter fine-tuned SARS-CoV-2 RBD heavy chain classifier based on the 6-layer ESM-2 8M backbone with a single-label sequence-classification head. We refer to this as the initial Model 1 (see Methods, and Supplementary Information, Model Development). Model 1 exceeded 80% classification accuracy on the test dataset, establishing the feasibility. We then implemented systematic parameter-efficient fine-tuning by Low-Rank Adaptation (LoRA) (51), which modifies only a small fraction of model weights while preserving the pretrained knowledge encoded in ESM-2’s 8M and 650M parameters (see Methods). Hyperparameters were optimized through systematic searches with two objectives: maximizing F1-score (model ASPred-F1) and minimizing validation loss (model ASPred-VL), yielding two model variants for each antigen. Stratified 3-fold cross-validation on held-out test sets performed consistently across antigens: accuracy with heavy-chain sequences was 80–88%, with AUROC values of 0.88–0.92 and F1-scores of 0.82–0.86 (Fig. 2A–C; Supplementary Fig. S1; Supplementary Data 2). Performance with heavy-chain–only inputs was comparable in magnitude to models trained on concatenated heavy–light pairs (Fig. 2A–C; Supplementary Fig. S1; Supplementary Data 2), consistent with heavy-chain sequences alone encoding sufficient information for specificity prediction.

To assess whether fine-tuning improved upon pretrained representations, we compared antibody sequence embeddings before and after training using several dimensionality-reduction methods (t-SNE, UMAP, and PCA). Across all three antigens and both optimization objectives, ASPred-F1 and ASPred-VL embeddings exhibited consistently higher silhouette scores and lower Davies–Bouldin indices than pretrained embeddings, indicating improved separation between antigen-specific and non-specific antibodies (Fig. 2D; Supplementary Data 3; all *p* < 0.001). As an additional baseline focused on the SARS-CoV-2 RBD target-specific antibody prediction task, we trained a regularized logistic regression classifier on fixed embeddings from AbLang (52), the current standard, using the same heavy-chain SARS splits as in ASPred. This baseline achieved 55.2% ± 0.9% accuracy on our data across 10-fold cross-validation (Supplementary Fig. S2). Thus, ASPred substantially improved separability on the same task, indicating that task-adaptive fine-tuning of large PLM representations is critical for antigen-specificity prediction from heavy-chain sequence.

### ASPred predictions align with experimental antibody mapping in human repertoires

**Figure 3:**
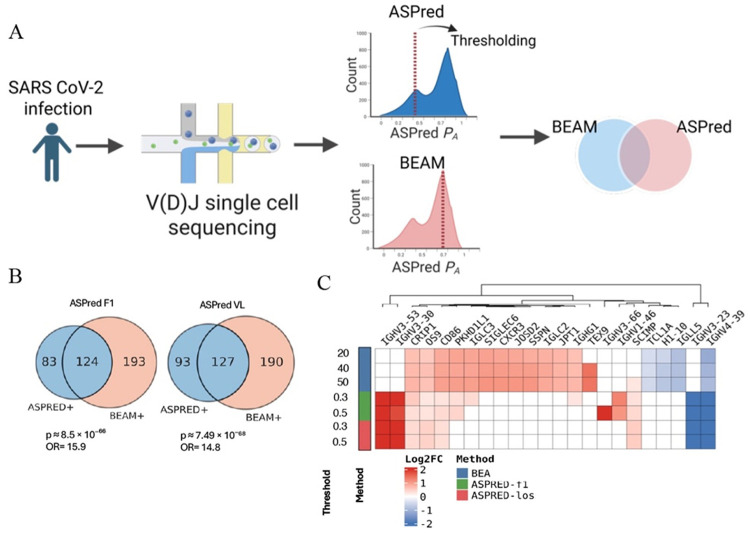
Benchmarking ASPred against BEAM. **(A)** Schematic of benchmarking ASPred predictions against the BEAM single-cell dataset from SARS-CoV-2-infected individuals. **(B)** Overlap between sequences predicted by ASPred (AS^+^) and experimentally discovered by BEAM technology (BEAM^+^) at fixed thresholds for ASPred-F1 and ASPred-VL (Total candidate BCR sequences: n =2,464). **(C)** Concordance between upregulated gene signatures in cells expressing the corresponding BCR V(D)J sequences across BEAM and ASPred thresholds (hierarchically clustered heat map of enrichment scores). The data are from 10x Genomics scRNA-seq and V(D)J sequencing, from sorted PBMC cells challenged with RBD. BEAM scores of 20, 40, and 50 were used as cutoffs for admitting cells as antigen-specific (BEAM^+^); similarly, the same BCR sequences from V(D)J reads had ASPred scores (P_A_) above cutoffs of 0.3 or 0.5, respectively, mRNA expression was quantified as UMI counts and log-normalized for downstream analysis.

To benchmark ASPred against established experimental methods, we analyzed a dataset in which B cells from SARS-CoV-2-infected individuals were profiled by single-cell RNA sequencing with paired V(D)J sequencing and BEAM technology (20)—a high-throughput microfluidic assay that ranks BCRs by RBD-binding propensity (BEAM score 0–100) (Fig. 3A).

This previously unseen dataset (provided by 10x Genomics, Supplementary Data 4) was comprised of 2,464 BCR sequences with both BEAM scores and complete heavy-chain V(D)J annotations, enabling direct comparison between computational and experimental antigen-specificity assignments. We applied ASPred to predict RBD-specificity for each sequence, generating probability scores. Using ASPred antigen-specificity score threshold (*P*_*A*_) ≥ 0.5 to classify sequences as antigen-specific (or AS^+^, in contrast to antigen-nonspecific or AS^−^), we observed highly significant overlap of AS^+^ with BEAM-positive calls (BEAM score ≥ 50): 124 sequences were identified by both methods (*p* = 8.5 × 10^− 66^, odds ratio = 15.9, Fisher’s exact test; Fig. 3B). ASPred identified 207 unique positives compared to 317 by BEAM. This observation likely reflects fundamental differences between the two methods: BEAM measures *in vitro* binding under defined artificial conditions, whereas ASPred learns high-dimensional sequence patterns associated with antigen recognition across diverse training examples and conditions.

To investigate whether ASPred predictions reflected biological signal, we leveraged paired transcriptomic data to compare gene expression profiles of B cells classified as antigen-specific. Differential expression analysis of cells predicted to display antigen-specific BCRs by ASPred-VL with a threshold *P*_*A*_ ≥ 0.5 revealed that AS^+^ cells exhibited upregulation of *IGHV3–53* (log_2_ FC = 2.54, *p*_adj_ = 3.20 × 10^− 19^)—a gene segment in B cells previously associated with SARS-CoV-2 neutralizing antibodies (53–55) (Fig. 3C)—as well as immune activation markers including *SCIMP* (Fig. 3C; Supplementary Data 5). Notably, these transcriptional signatures were enriched in ASPred-predicted AS^+^ cells regardless of BEAM classification, suggesting that ASPred identifies B cells with transcriptomic hallmarks of antigen experience. Conversely, varying the BEAM threshold from 20 through 40 to 50 showed consistent trends in differential gene expression patterns, with strongest signals at higher BEAM cutoffs (Fig. 3C). These results together suggest that ASPred-predicted antibodies achieve high biological relevance.

### ASPred identifies antigen-specific BCRs in unselected mouse immune repertoires

**Figure 4:**
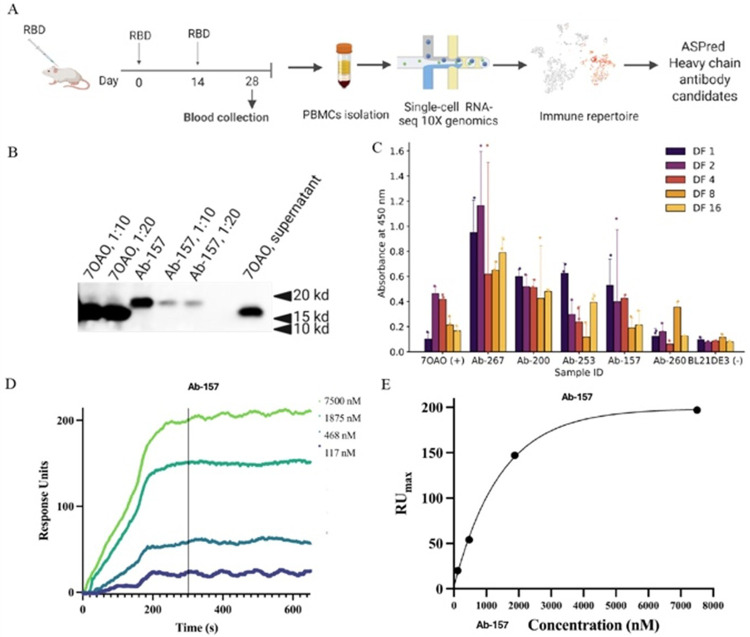
Experimental validation of ASPred-predicted antibodies from single-cell repertoire analysis. **(A)** Experimental workflow: Mice were immunized, and single peripheral blood mononuclear cells (PBMC) were isolated by 10x Genomics Chromium chip, barcoded and scRNAseq/V(D)J sequences were obtained, filtered and analyzed as described in Methods. The V(D)J heavy sequences were analyzed by ASPred to provide a specificity score. **(B)** Western blot confirming expression of selected VHHs [see Supplementary Fig. 4 for the entire gel blot]. **(C)** ELISA binding assays of ten randomly chosen VHH candidates from ASPred outputs across serial dilutions (mean ± s.e.m.) to SARS-CoV-2 RBD. Reference antibody was nanobody C5 (56) labeled here as 7OAO. DF1, DF2, DF4, DF8, and DF16 are dilution factors 1x, 1/2 x, 1/4 x, 1/8 x, and 1/16 × of the antibodies, respectively. **(D, E)** LSPR sensorgrams of purified VHHs; Ab-157 shows high-affinity binding (K_D_=20.7 nM).

To evaluate ASPred’s capacity to discover novel functional antibodies from whole immune repertoires without experimental pre-selection, we immunized BALB/c mice with SARS-CoV-2 RBD and performed single-cell RNA sequencing with V(D)J junction sequence profiling on unsorted peripheral blood mononuclear cells (PBMCs) 28 days post-immunization (Fig. 4A). This experimental design intentionally avoided antigen-tagged sorting or enrichment, generating an unbiased snapshot of the circulating BCR repertoire. After quality filtering, we obtained 312 unique heavy-chain V(D)J sequences representing diverse B cell clonotypes.

Applying ASPred Model 1 (*P*_*A*_ ≥ 0.5), we classified 29 BCR sequences as putatively RBD-specific (Supplementary Fig. S3; Supplementary Data 6). To assess predictive accuracy, we randomly selected 10 candidates out of 29 spanning a range of ASPred scores (*P*_*A*_ = 0.59–0.99) for experimental validation. Their corresponding DNA sequences were codon-optimized, synthesized, and cloned. We successfully expressed 5 of these as variable heavy domain (VHH) nanobodies in *E. coli*. Following periplasmic extraction and initial screening by anti-His immunoblots (Fig. 4B), we tested binding to recombinant RBD by enzyme-linked immunosorbent assay (ELISA). All five candidates demonstrated concentration-dependent binding to RBD at affinities comparable to the commercially obtained reference nanobody C5 (56) (Fig. 4C), representing a validation rate that is substantially above the expected baseline prevalence of antigen-specific BCRs (0.005–0.01% of unselected immune repertoires) (1, 2).

We successfully purified one strongly AS^+^ candidate antibody Ab-157, which exhibited high-affinity binding to immobilized RBD by localized surface plasmon resonance (LSPR), with kinetic parameters *K*_*D*_ = 20.7 nM, *k*_on_ = 3.25 × 10^3^ M^−1^s^− 1^, and *k*_off_ = 6.72 × 10^− 5^ s^− 1^ (Fig. 4D,E). A second purified candidate antibody Ab-200 also showed concentration-dependent binding to RBD by ELISA (Supplementary Fig. S5). These results establish that candidates with high ASPred scores are enriched for functional RBD-binding antibodies from unselected immune repertoires.

### Predicted antigen-specific sequences exhibit distinct structural and evolutionary signatures

To characterize the sequence properties of ASPred-identified BCRs, we integrated V(D)J sequence data with paired single-cell transcriptomes from the RBD-immunized mouse repertoire described above. Unsupervised clustering and cell-type annotation based on marker gene expression (Methods) revealed that most AS^+^ sequences originated from mature B cells (Supplementary Data 7), consistent with antigen-driven selection and activation. A small fraction mapped to lambda-expressing pre-B-like cells, potentially reflecting reference mapping artifacts given the rarity of pre-B cells in peripheral blood.

Differential gene expression analysis comparing AS^+^ against AS^−^ B cells identified the upregulation of chemokine transcript CCL5 (log_2_ FC = 5.28, *p*_adj_ = 1.45×10^− 5^), cytotoxicity marker transcript NKG7 (log_2_ FC = 5.47, *p*_adj_ = 0.011), and leukocyte-specific transcript LST1 (log_2_ FC = 4.09, *p*_adj_ = 0.0019) in cells predicted to express RBD-specific BCR (Supplementary Table S2). These observations suggest recent immune activation and T cell-B cell collaboration, supporting the biological plausibility of ASPred classifications.

**Figure 5:**
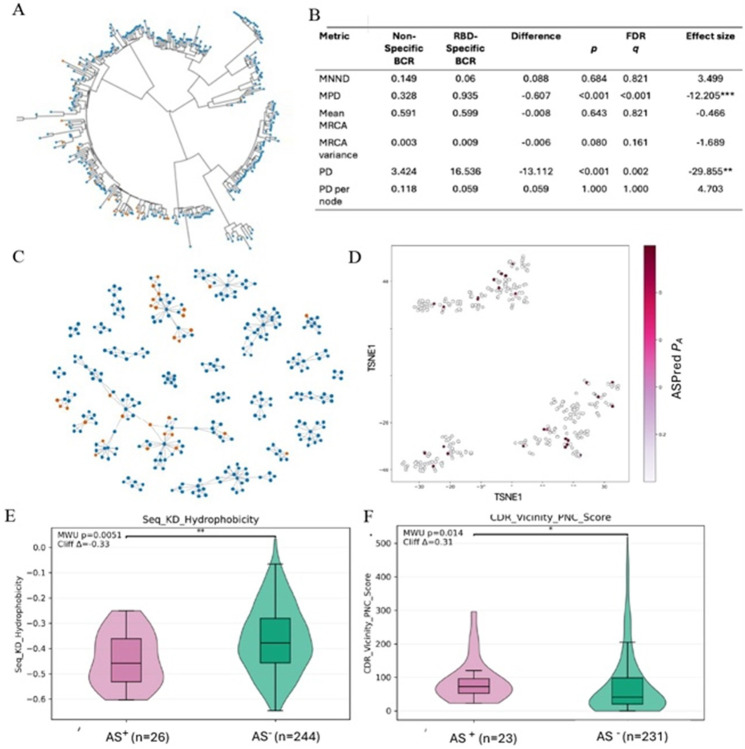
Structural and evolutionary features of ASPred-predicted antigen-specific antibody sequences. **(A)** Maximum likelihood tree alignment of VHH sequences of the whole immune repertoire B cells (1,000 bootstraps). V(D)J sequences were trimmed (see Methods). Red, AS^+^; blue, AS^−^ BCR sequences, respectively. **(B)** Measures of phylogenetic diversity of the B cell receptor repertoire. We measured phylogenetic sequence diversity by mean nearest-neighbor distances (MNND), mean and variance of the node-to-most-recent common ancestor (MRCA) distances, patristic distances (PD), and PD per node (see Methods). **(C)** K-nearest neighbor (k=3) network of the AS^+^ and AS^−^ BCR sequences. Edges represent the nearest neighbors. Red, AS^+^ BCRs; blue, AS^−^ BCRs. **(D)** Low-dimensional sequence-space embedding showing AS^+^ BCRs (magenta) and AS^−^ BCRs (gray) dispersed across clusters. **(E)** Violin/box plots of sequence hydrophobicity (Kyte–Doolittle GRAVY); distributional shift between AS^+^ BCRs and AS^−^ BCRs (two-sided Mann–Whitney U; Source Data). (**F**) Violin/box plots of CDR-vicinity Patches of Negative Charge (PNC); AS^+^ BCRs differ from AS^−^ BCRs (two-sided Mann–Whitney U; exact P in the panels).

Phylogenetic analysis of the 312 heavy-chain sequences thus identified revealed that AS^+^ BCRs occupied distinct regions in the antibody sequence space. As anticipated, maximum likelihood trees with 1,000 bootstrap replicates showed significant clustering of predicted RBD-specific sequences (Fig. 5A). Consistent with the idea that AS^+^ BCRs are more related to one another, the mean pairwise phylogenetic distance (MPD = 0.08 vs 0.19 for AS^−^; *q* < 0.01, permutation test) and total phylogenetic diversity (PD = 2.4 vs 5.8; *q* < 0.01; Fig. 5B) were both reduced in the antigen-specific classes. *k*-nearest neighbor network analysis (*k* = 3) demonstrated that predicted RBD-specific sequences formed interconnected clusters with somewhat higher clonality than the background sequences (Fig. 5C), while t-SNE projections showed dispersion across multiple clusters rather than a single tight group (Fig. 5D), indicating that ASPred captures diverse sequence-level solutions to RBD recognition rather than a single dominant clonotype.

To identify properties that distinguish the predicted binders, we computed biophysical features using homology models generated by IgFold (57). AS^+^ sequences exhibited significantly elevated CDR-proximal hydrophobicity (Kyte-Doolittle GRAVY (58); median = 0.32 vs 0.21, *p* = 0.005, Mann–Whitney *U*; Fig. 5E) and larger patches of negative surface charge near CDR regions (patch of negative charge; PNC; median = 48 vs 35Å^2^, *p* = 0.014; Fig. 5F). These properties align with features of the RBD binding interfaces of known antibodies against RBD, which present both hydrophobic residues (F486, Y489, L455) and a negatively charged surface patch (E484, D405, D420) (59), suggesting that ASPred learns biochemically relevant recognition patterns.

### Molecular simulations support predicted antibody–RBD interactions

**Figure 6:**
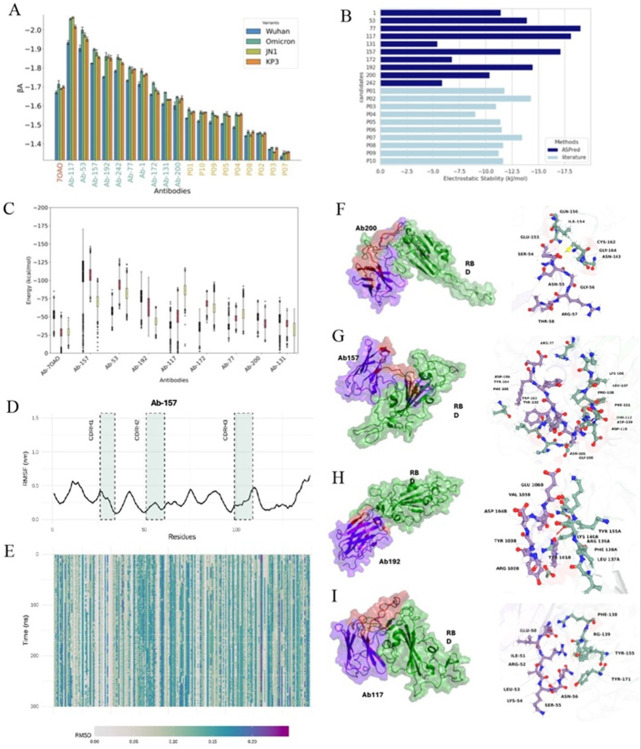
Multiscale in silico validation of ASPred-predicted VHH–RBD interactions. **(A)** Constant-pH coarse-grained simulations estimating affinity scores (β_A_) for four RBD variants (Wuhan, Omicron, JN.1, KP.3); bars show mean±s.e.m. **(B)** Electrostatic stability of ASPred-predicted VHHs. **(C)** Binding free energies from explicit-solvent MD using MM-PBSA for predicted and positive control (reference nanobody C5 (56) labeled here as 7OAO) for binding to the Wuhan RBD only; box plots summarize replicate trajectories per antibody. **(D)** Stability analysis for Ab-157 across 300ns MD. (**E**): per-residue RMSF with CDR windows indicated; bottom: residue-wise RMSD heat map over time showing a stable paratope–epitope interface. **(F-I)** Representative complexes for four antibodies (Ab192, Ab200, Ab117, Ab157). Left: RBD (green surface) with VHH (purple cartoon; CDRs in red). Right: interface zooms highlighting recurrent hydrogen bonds and salt bridges.

To investigate binding mechanisms at molecular resolution, we performed multi-scale computational modeling of ASPred-predicted antibodies in complex with SARS-CoV2 RBD. Heavy-chain structures were generated using IgFold (57) and docked to RBD (AlphaFold2 model) using ClusPro 2.0 (60) with antibody-specific constraints. For the ten experimentally tested candidates described above, we selected top-ranked docking poses and estimated binding energies using PRODIGY (61). Several predicted binders, including Ab-157 and Ab-200, showed favorable binding energies comparable to the control nanobody C5 (*PDB*: 7OAO) (56), a well-characterized RBD binder (Supplementary Fig. S6). We next employed constant-pH coarse-grained Monte Carlo molecular dynamics simulations (62) to estimate binding free energies under physiological conditions (pH 7.4, 150 mM NaCl). All ASPred-predicted candidates exhibited favorable binding profiles (free energy, *β* *A* < − 5*k*_*B*_*T*), with Ab-117, Ab-53, and Ab-157 showing the strongest predicted affinities (Fig. 6A).

Interestingly, these three antibodies maintained favorable binding predictions against emerging SARS-CoV-2 variants (Omicron BA.1, JN.1, KP.3), suggesting potential cross-reactivity (Fig. 6A). Electrostatic stability analysis using Fast Proton Titration Scheme (FPTS) (63) indicated that ASPred-predicted antibodies exhibited stability profiles similar to or exceeding known anti-RBD antibodies (Fig. 6B).

To probe atomic-level interactions, we performed explicit-solvent molecular dynamics simulations with GROMACS (64) (version 2024.3) using the AMBER99SB-ILDN force field (Methods) for eight ASPred-predicted VHH–RBD complexes over 300 ns trajectories with three independent replicates each. Complexes remained structurally stable with low backbone RMSD (< 3Å) and maintained persistent CDR–epitope contacts throughout simulations. Binding free energies computed via Molecular Mechanics Poisson–Boltzmann Surface Area (MM-PBSA) (65) showed that ASPred-predicted antibodies achieved affinities comparable to the C5 control (7OAO), with Ab-157—experimentally validated at *K*_*D*_ = 20.7 nM—displaying particularly stable interactions (Fig. 6C). Per-residue energy decomposition highlighted key contributions from CDRH3 residues forming hydrogen bonds and salt bridges with RBD epitope residues (Fig. 6D,E), providing mechanistic insight into molecular recognition. Residue-level interface analysis of representative complexes indicated that binding was consistently dominated by CDR-centered recognition surfaces, particularly CDR3, although the detailed contact chemistry varied across antibodies (Fig. 6D,E; see Supplementary Information). For clarity, Figs. 6D and E show four representative complexes (Ab-192, Ab-157, Ab-117, and Ab-200) chosen to illustrate distinct interface architectures observed across the modeled set (see Supplementary Information for additional structural details).

Ab-157 formed a broad, chemically balanced interface supported by a network of 15 hydrogen bonds and 2 salt bridges, with many antibody-side contacts concentrated in the 98–120 segment, consistent with a dominant CDR3 contribution. Ab-117 likewise formed an extensive interface, comprising 12 hydrogen bonds, 1 salt bridge, and 1 *π*–*π* interaction, again with major contributions from the 104–126 region and additional contacts from other loop regions. In contrast, Ab-200 adopted a smaller but well-packed interface with no clashes, 8 hydrogen bonds, and a more nonpolar contact profile, with interactions concentrated in residues 109–123 region and reinforced by additional contacts from other CDR-like segments. Ab-192 similarly exhibited a multi-contact binding mode in which residues around Tyr101–Glu106 formed a dominant CDR3-centered recognition patch, supported by additional contacts from CDR1- and CDR2-like segments.

Together, these simulations support the conclusion that ASPred-selected antibodies engage RBD through structurally diverse but recurrent CDR-dominated interfaces, providing a mechanistic basis for the experimentally observed enrichment of binders among high-scoring predictions.

### ASPred detects antigen-specific signatures in bulk-sequenced human repertoires

To assess whether ASPred detects antigen-driven responses in naturally infected individuals, we analyzed publicly available bulk heavy-chain BCR sequencing data from peripheral blood of SARS-CoV-2-infected patients (*n* = 24 individuals) and healthy controls (*n* = 10 individuals) (Supplementary Data 8). These repertoires, generated without single-cell resolution or antigen enrichment, contained ~10^4^–10^5^ sequences per individual. We scored all sequences with ASPred and stratified each repertoire into predicted antigen-associated (AS^+^; *P*_*A*_ ≥ 0.5) and non-specific (AS^−^; *P*_*A*_ < 0.5) subsets.

**Figure 7:**
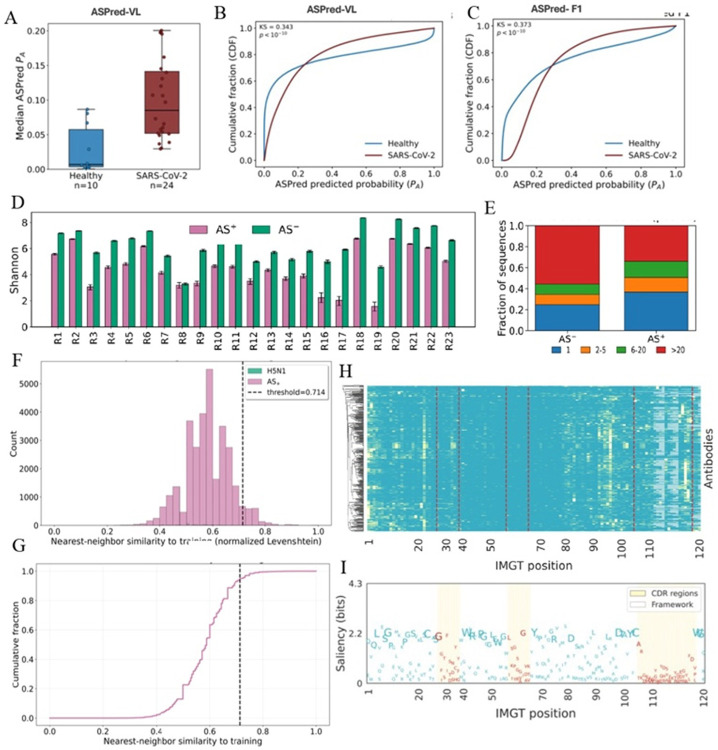
Diversity and training–data relatedness of ASPred^+^ BCRs in bulk repertoires. **(A)** Per-sample median ASPred predicted antigen-association probability (P_A_) for healthy (blue; n = 10) and SARS-CoV-2 (red; n =24) repertoires using ASPred-VL. Mann-Whitney p = 1.4×10^−3^. **(B, C)** Empirical CDFs of predicted probabilities (P_A_) comparing healthy and SARS-CoV-2 repertoires for ASPred-VL (**B**) and ASPredF1 (**C**); Kolmogorov–Smirnov statistics are shown. **(D)** Per-donor clonal diversity (Shannon entropy, H) for AS^+^ (magenta) and AS^−^ (green). Bars show mean with 95% bootstrap CIs (1000 resamples). Paired t-test across all 23 repertoires were significant to p < 0.05 (Supplementary Data 9). **(E)** Clone-size composition (pooled across donors) for AS^+^ and AS^−^, showing the fraction of reads in bins 1, 2–5, 6–20, and > 20 members. Each AS^+^ clone size was significantly different (p<0.001) from that of AS^–^ clone size in the respective bin. **(F, G)** Novelty of the fine-tuning training set quantified by nearest-neighbor normalized Levenshtein similarity of AS^+^ sequences to training examples: histogram (**F**) and empirical CDF (**G**). The vertical line indicates a negation-derived threshold set as the 99th percentile of similarities from an unrelated influenza (H5N1) control repertoire (δ =0.01; summary statistics in Supplementary Table S3). Corresponding novelty plots for ASPred-VL are shown in Supplementary Fig S8. **(H, I)** Saliency of ASPred predictions across the international ImMunoGeneTics information system (IMGT) aligned positions: per-sequence saliency heat map (**H**) and aggregate sequence logo (**I**), with CDR regions highlighted, showing distributed contributions across multiple CDRs rather than a small set of conserved positions.

At the repertoire level, SARS-CoV-2–infected individuals exhibited significantly higher median ASPred probabilities than healthy controls (two-sided Mann–Whitney *U* test; Fig. 7A), consistent with a donor-level enrichment of high ASPred-scoring BCRs. At the sequence level, ASPred probability distributions were right-shifted in infected individuals, with significant separation by Kolmogorov–Smirnov test in pooled CDFs (ASPred-VL: KS = 0.343; ASPred-F1: KS = 0.373; *p* < 10^− 10^; Fig. 7B,C), consistent with enrichment of antigen-associated BCRs during infection.

To evaluate sequence diversity, we computed Shannon entropy (*H*) (66, 67) and inverse Simpson index (1*/D*) (68) for AS^+^ and AS^−^ subsets using equal-depth resampling to control for coverage differences. AS^+^ sequences exhibited significantly lower diversity than AS^−^ across all infected repertoires, with non-overlapping 95% bootstrap confidence intervals at the donor level (Fig. 7D), reflecting clonal focusing characteristic of antigen-specific responses. Clone-size composition (pooled across donors) was consistent with reduced diversity in AS^+^: AS^−^ repertoires were dominated by highly expanded clones (> 20 members: 55.8% vs. 33.7% in AS^+^; Fig. 7E), whereas AS^+^ sequences were comparatively shifted toward smaller clone-size bins (significant *p* < 0.001).

Phylogenetic analysis of 100 synthetic datasets (each containing 127 randomly sampled AS^+^ and 127 AS^−^ sequences) revealed significant clustering of AS^+^ sequences on maximum likelihood trees (median Fitch parsimony *Z*-score = − 13, *p* < 0.001 vs permutation null; Supplementary Fig. S7a,b), indicating that predicted antigen-specific antibodies share recent common ancestry. Local enrichment analysis showed that AS^+^ sequences predominantly neighbor other AS^+^ sequences (mean *k* = 3 nearest-neighbor fraction = 0.89; Supplementary Fig. S7c,d), supporting convergent sequence evolution toward RBD recognition.

### Predicted sequences show minimal similarity to training data

A critical concern for machine learning models is whether predictions reflect genuine pattern recognition or merely memorization of training examples. To address this, we quantified the similarity between ASPred-predicted RBD-specific (AS^+^) sequences from human repertoires and all sequences in the training dataset. For each AS^+^ sequence, we computed the maximum normalized Levenshtein similarity (68) to any training sequence with matching CDRH3 length, approximating the Hamming distance (69).

The distribution of maximum similarities showed that most AS^+^ sequences (93.8%) fell below the negation-derived novelty threshold with a median similarity of 0.588 (Fig. 7F). To establish a threshold for “novel” sequences, we computed the 99th percentile of similarity scores for a negative control set (antibodies targeting unrelated influenza antigens), yielding a cutoff of 0.714 (*δ* = 0.01; Fig. 7G, dashed vertical line). By this conservative criterion, 93.8% of AS^+^ predictions qualified as novel relative to training data, indicating that ASPred predictions are not driven by the memory of training examples.

Consistent with this conclusion, saliency analyses across IMGT-aligned positions [IMGT is the international ImMunoGeneTics information system, which provides a standardized numbering scheme for antibody variable domains (70)] demonstrated that ASPred predictions depend on distributed sequence features spanning multiple complementarity-determining regions rather than a small number of highly conserved positions (Fig. 7H, I) (full set of IMGT-mapped saliency plots provided in Supplementary Data 9). Together, these results support the conclusion that ASPred generalizes learned antigen-specific patterns beyond simple sequence similarity to its training data.

## Discussion

We have shown that protein language models can be fine-tuned to infer antigen specificity from heavy-chain sequence alone, and we validated this capability across three viral antigens and multiple biological settings. Heavy-chain-only models perform as well as, sometimes better than, models using paired heavy–light inputs. This does not negate the well-established role of light chains in modulating affinity and fine specificity (48, 72) but reemphasizes that heavy chains—particularly features concentrated in and around CDRH3—often contains significant information to encode specificity (73, 74). These results also address a fundamental question in adaptive immunity: how much functional information is encoded in antibody sequence versus its evolutionary history and other related cell biological contexts? The success of sequence-only prediction of ASPred suggests that evolution has embedded a remarkable extent of specificity information in the heavy chain, though context (somatic hypermutation history, clonal expansion, transcriptional state of the cell) clearly would add important dimensions.

An important concern for computational repertoire classifiers such as ASPred is whether they memorize overrepresented motifs or whether they genuinely learn the contextual and functional grammar, and thus are capable of true generalization. This is a difficult point to settle. However, several observations support genuine generalization by ASPred. First, predicted binders in bulk human repertoires are largely distinct from the training sequences by nearest-neighbor similarity criteria. Second, predicted antigen-specific subsets show repertoire-level markers of immune response—right-shifted specificity scores in infected versus healthy individuals, elevated clonality, and restricted phylogenetic structure—features that are difficult to explain by simple template matching. Third, predicted binders display interpretable biophysical signatures, consistent with known properties of the RBD interface. The phylogenetic dispersion of predicted binders further supports the idea, although does not prove, that ASPred captures diverse structural solutions to antigen recognition, not the clonal expansion of a small number of sequence families. We suspect that this generalization capacity likely emerges from ESM-2’s large-scale pretraining on natural protein sequences, which evidently encode high-dimensional evolutionary patterns, and biophysical and structural contingencies. Fine-tuning adapts these high-dimensional representations to the specific task of antigen recognition, effectively transferring the general protein “knowledge” to the specialized domain of antibody function.

Several important caveats should be noted. First, ASPred’s training data, derived primarily from curated antibody databases, inherently over-represents functional binders and lacks comprehensive true negatives—non-binding sequences with experimental evidence of absence of binding, rather than the absence of evidence. Addressing this issue will require systematic generation of experimentally validated negative datasets (75) or semi-supervised learning approaches that leverage the vast unlabeled repertoire space. Second, while heavy-chain–only prediction enables analysis of bulk sequencing data, incorporating paired light chain information might improve accuracy and enable prediction of binding affinity, not just specificity. The current approach trades completeness for scalability—a reasonable compromise for initial screening, but refinement should benefit from paired-chain architectures. Third, our *de novo* validation used a single immunization model and limited antigen diversity. Extending to diverse epitopes, antibody formats (e.g., fully human vs. murine), and isotypes will test true generalizability, although its discriminatory power is well supported by its success across murine and human immune repertoires. The model’s transferability to non-viral antigens, tumor-associated antigens, or self-antigens remains to be established.

The highly significant overlap between ASPred and BEAM positives supports biological validity; moderate concordance is expected because the two methods operationalize “specificity” differently. BEAM directly measures binding *in vitro* under defined antigen presentation, avidity, and kinetic regimes; it can miss antibodies that depend on native conformations, particular glycoforms or contextual presentation, and it may flag as binders some that are weak, polyreactive, or those that are sensitive to assay conditions. ASPred, in contrast, learns sequence patterns associated with antigen recognition across diverse labeled examples; it may recover antibodies sharing recognition “grammar” even when their binding kinetics place them near the detection boundary of an experimental assay. The enrichment of antigen-associated IGHV usage and immune activation signatures among ASPred-positive cells—even when BEAM scores are below the chosen cutoff—suggests ASPred may be sensitive to biologically meaningful antigen experience beyond a single binding threshold. These considerations argue for a complementary strategy: experimental mapping validates and calibrates small sets at high confidence, while ASPred enables repertoire-wide discovery, retrospective analysis of archived samples, and hypothesis generation at scale.

For therapeutic antibody discovery, mining whole immune repertoires offers intrinsic advantages over synthetic or display-based methods. Naturally selected antibodies have survived *in vivo* tolerance checkpoints, exhibit appropriate folding and expression, and lack many developability-related issues (76) that plague library-derived candidates often have. ASPred provides a computational lens to focus on the most promising sequences within this natural diversity.

Predicting antigen specificity directly from repertoire sequencing may convert abundant V(D)J mRNA sequence datasets, especially from those enriched for sequences near the 5’ ends (71), into functional hypotheses—an enabling capability for antibody discovery, vaccine evaluation, and immune surveillance. In practice, bulk 5’-enriched or V(D)J-only deep-sequence datasets can now become accessible to computational screening, with paired-chain and biophysical characterization reserved for downstream refinement. The observed “hit rate” of ASPred, even allowing for difficulties in developability of as many as 50% of the candidate antibodies, represents orders-of-magnitude enrichment over the expected frequency of antigen-specific BCRs in unselected repertoires. It is unclear at this time, however, whether a general model is feasible, which can identify specific antibodies to any arbitrary antigen—this remains a considerable theoretical and technical challenge. Future integration with structure prediction and generative design (77) could enable a closed loop: predict binders, model their structures, optimize affinity *in silico*, and validate experimentally. Coupling with single-cell transcriptomics should reveal cellular contexts—identifying which B cell subsets produce the most promising candidates. As immune repertoire databases grow, particularly longitudinal datasets capturing vaccination and infection responses, ASPred-like models promise to become increasingly powerful for understanding immune memory, vaccine efficacy, and pathogen evolution.

To facilitate broader use, we provide a public web interface for ASPred at https://ASPred.org/, enabling probability scoring of heavy-chain sequences without local computing.

## Materials and Methods

### Experimental Design

Dataset curation, design of computational workflow and model optimization strategies are schematically explained in Fig. 1A. Model testing and validation are diagrammatized in Fig. 1B,C. Here we describe the specific methods in detail

### SARS-CoV-2 RBD dataset curation for the initial Model 1 classifier

For the initial SARS-CoV-2 heavy-chain classifier used for prospective scoring of the mouse repertoire (Model 1), we curated a supervised dataset containing experimentally validated binders and a matched non-target set. Positive examples were obtained from CoV-AbDab and consisted of heavy-chain variable-region (VH) sequences annotated to bind SARS-CoV-2 receptor-binding domain (RBD). Negative examples were derived primarily from PLAbDab entries not annotated to coronavirus-family targets.

To improve data quality, sequences with incomplete variable regions or very short length were removed, and exact duplicate VH amino-acid sequences were collapsed. For split hygiene, we further required recoverable CDRH3 boundaries and used CDRH3-based similarity clustering to reduce near-duplicate leakage between train, validation, and test partitions. Under this strategy, all sequences assigned to the same clonotype-proxy cluster were placed in the same partition. Additional dataset audit summaries and split-hygiene details for Model 1 are provided in the Supplementary Information.

### Models and data sources

We evaluated two pretrained protein language models based on the ESM-2 architecture: ESM2-t6-8M and ESM2-t33-650M (facebook/esm2_t6_8M_UR50D and facebook/esm2_t33_650M_UR50D) for antigen-specificity prediction. A binary classification head was attached to each backbone, and sequences were tokenized with the corresponding ESM tokenizer.

Training datasets were assembled from publicly available antibody repositories. For SARS-CoV-2 receptor-binding domain (RBD), influenza hemagglutinin (HA), and HIV gp120, we collected antigen-specific antibody sequences from CoV-AbDab and related curated sources, using heavy-chain, light-chain, or concatenated heavy–light chain inputs as indicated for each model. Negative examples were defined as antibody sequences annotated outside the target antigen class, including non-target entries from PLAbDab and related datasets, while maintaining balanced class ratios for training and evaluation (31, 50, 78).

Model development was performed using stratified 3-fold cross-validation. The best-performing configurations were then retrained on the full training datasets and evaluated on held-out test sets. Per-fold performance summaries are provided in Supplementary Data and Supplementary Information, and full model configuration details are given in Supplementary Table S1.

### Initial model

We performed an end-to-end initial fine-tune of the 6-layer ESM-2 8M mode (facebook/esm2_t6_8M_UR50D) using a single-label classification head (EsmForSequenceClassification). Inputs were tokenized with the ESM tokenizer (EsmTokenizer; vocabulary size 33) and padded/truncated to 1,024 tokens. Model hyperparameters followed the saved configuration (config.json): hidden size 320, intermediate size 1,280, 20 attention heads, GELU activations, layer norm *ϵ* = 10^− 5^, hidden dropout = 0.1, attention dropout = 0.0, rotary position embeddings (max positional capacity = 1,026), and output_hidden_states=true; training used FP32 under transformers v4.32.1. Trained model is provided as Supplementary Data 10. Subsequently, we performed fine-tuning by systematic statistical methods as described below.

### Parameter-efficient fine-tuning (PEFT/LoRA)

The fine-tuning used LoRA adapters inserted into each block’s self-attention projections (**W**_*q*_,**W**_*k*_,**W**_*v*_), with all other backbone weights frozen and the classification head trainable. We ran Optuna hyperparameter optimization under stratified 3fold cross-validation in two complementary modes: (1) an F1-maximization study with search ranges lora_r ∈ {4,8}, lora_alpha ∈ [16,64], lora_dropout ∈ [0.05,0.10], learning rate [5 × 10^− 7^, 2 × 10^− 5^] (log-uniform), batch size {4,8}, epochs [4,8], and warmup ratio [0.05,0.20], trained via transformers. Trainer with early stopping (patience = 1), fp16 when CUDA was available, gradient accumulation (2 steps), pinned dataloaders, and selection by mean F1 across folds; (2) an explicit validation-loss minimization study that optimized eval_loss by setting metric_for_best_model to eval_loss and greater_is_better to False, widened certain ranges (lora_r ∈ {4,8,16}, lora_dropout ∈ [0.05,0.30], learning rate [10 − ^6^, 5 × 10 − ^5^], epochs [2,6], warmup ratio [0.05,0.20], batch size {4,8}), and returned the negative average fold loss to keep a maximize objective. Both studies logged per-fold accuracy, precision, recall, F1, ROC–AUC, confusion matrices, ROC curves, and artifacts to Weights & Biases with timestamped project names, and after HPO we retrained the selected backbone on the full dataset with best hyperparameters, exported PEFT-augmented checkpoints, and saved the Optuna studies (.pkl) plus optimization-history and parameter-importance plots under a versioned directory structure. Evaluation reported F1 as primary along with accuracy, precision, recall, ROC–AUC, and confusion matrices computed with scikit-learn, deriving ROC from softmax positive-class probabilities and writing per-fold predictions (label, pred, prob) to CSV. Jobs were executed on an HPC cluster under Slurm using L40S GPUs (typically 2× L40S, 120GB RAM, 20 CPU cores) with environment modules gcc/13.3.0, mpich/4.2.2, and a Conda environment containing pytorch, transformers, peft, optuna, and wandb.

### Animal Experimentation Ethics statement

All animal procedures were approved by the University of California, Riverside Institutional Animal Care and Use Committee (IACUC) and conducted in accordance with institutional and federal guidelines (protocol #AUP20210029).

### Immunization and sample collection

Mice were housed in the University of California, Riverside vivarium. Six-week-old female BALB/c mice were immunized by subcutaneous injection in the back of the neck on days 0 and 14 with 100 *μ*L of SARS-CoV-2 RBD antigen emulsified in 2% aluminum hydroxide. Blood samples were collected on days 14 and 28 after immunization. On day 28, mice were deeply anesthetized with isoflurane and blood was collected by cardiac puncture. Mice were then euthanized by cervical dislocation in accordance with IACUC guidelines. Peripheral blood mononuclear cells (PBMCs) were isolated using the direct human PBMC isolation kit (StemCell Technologies, Inc.) and cryopreserved at −80°C until further use.

### GEM Generation and construction of next-generation sequencing libraries

Cryopreserved peripheral blood mononuclear cells (PBMCs) from immunized mice were thawed rapidly at 37°C, transferred to pre-warmed complete growth medium, and centrifuged at 300 × g for 5 minutes to pellet cells. The cell pellet was resuspended in PBS supplemented with 0.04% BSA. Cell concentration and viability were determined using a manual hemacytometer with trypan blue exclusion, ensuring viability exceeded 85%. Cells were then adjusted to a final concentration of 700–1,200 cells/μL. Single-cell suspensions were mixed with nuclease-free water and 5′ single-cell RNA master mixture, then loaded into a Chromium chip with barcoded gel beads and partitioning oil. The chip was placed in the Chromium controller to generate gel beads in emulsion (GEMs). cDNA was obtained from 100 *μ* l GEMs/sample by reverse-transcription reactions: 53°C for 45 min, 85°C for 5 min, then maintained at 4°C. cDNA products were purified and cleaned using Dynabeads. cDNA was amplified by PCR: 98°C for 45s; 98°C for 20 s, 63°C for 30 s, 72°C for 1 min and amplified for 16 cycles; then, 72°C for 1 min. Amplified PCR products were purified using SPRIselect reagent kit (B23317, Beckman Coulter). The concentration of the cDNA library was determined by Qubit dsDNA HS Assay Kit (Invitrogen) and Bioanalyzer (Agilent, 2100) (Wang et al, 2023). Single Cell RNA-Seq V(D)J and 5’ gene expression library was performed using the Chromium Next GEM Single Cell 5’ Reagent Kits v2 (Dual Index)(CG000331, Rev E, 10X Genomics) and Dual Index kit TT set A (PN1000215, 10X Genomics) according to the manufacturer’s instructions. For unlabeled and unsorted samples, the target was estimated at 5000 cells.

### Identification of antigen-specific BCRs using InterClone

Clustering of sequences was performed using the source code of InterClone, which employed MMSeqs2 for clustering. The dataset being clustered was constructed with 11,917 known SARS-CoV-2 specific antibody heavy chain sequences from CovAbDab and 310 heavy chain sequences from our mouse RBD-immunized single cell heavy chains sequences. To identify antigen-specific B cell receptors (BCRs) from the total repertoire of peripheral blood mononuclear cells (PBMC), we employed three distinct approaches. The first approach utilized the clustering tool *InterClone* (79), which introduces a novel method to cluster antibodies sharing antigenic targets based on their complementarity-determining region (CDR) sequences, with *MMSeq2* facilitating effective sequence clustering through homology alignment and gap management. We constructed a dataset comprising 11,917 known SARS-CoV-2-specific antibody heavy chain sequences sourced from CovAbDab (50) and an additional 310 heavy chain sequences obtained from our own murine single-cell dataset. Using a clustering threshold of 70% for the CDR similarity index (SID) and requiring 90% coverage, we identified 96 candidate sequences from our single-cell testing dataset, which clustered with known SARS-CoV-2 antibody sequences.

### Cloning and recombinant VHH expression

DNA molecules of VHH sequences were synthesized by Integrated DNA Technologies (IDT). Each 20ng fragment was PCR-amplified using a New England Biolabs (NEB) kit. The pET-22b(+) vector (100ng) was linearized with *Not*I and *Nco*I, heat-inactivated at 80°C for 20 min, and assembled with 50 ng of PC-Ramplified fragments by Gibson assembly using the NEBuilder^®^ HiFi DNA Assembly Master Mix according to the manufacturer’s instructions (50°C for 60min). *E. coli* DH5*α*; transformants were screened by colony PCR and confirmed by Sanger sequencing. *E. coli* BL21 (DE3) competent cells were transformed with the plasmid constructs. Single colonies were cultured at 37°C to an OD_600_ of 0.6, induced with 1mM isopropyl *β*-D-1-thiogalactopyranoside (IPTG) at 37°C for 4h, and then incubated with agitation at 22°C overnight. Cultures were centrifuged, and cell pellets were resuspended in phosphate-buffered saline (PBS; 137mM NaCl, 2.7mM KCl, 10mM Na_2_HPO_4_, 1.8mM KH_2_PO_4_, pH7.4). Proteins were extracted by freeze–thawing twice. Samples were dialyzed overnight in 1L of PBS at 4°C. Protein expression was confirmed by dot blot using an HRP-conjugated anti-His antibody (Ay63, Cat. HIS-PLM535). As a positive control, a His-tagged S1S2 protein was used; negative controls included *E. coli* BL21 cells cultured without plasmid and Q*β* protein. Western blot analysis was performed by probing with the anti-His antibody; extracts of BL21 cells without plasmid served as the negative control. All constructs were expressed in *E. coli*, and a freeze–thaw protocol previously shown to efficiently release nanobodies into the culture supernatant (80) was used to obtain nanobody-rich supernatants for screening.

### ELISA assays of periplasmic VHH supernatants for SARS-CoV-2 RBD recognition

Following confirmation of VHH expression, antigen recognition was assessed by ELISA using unpurified periplasmic supernatants. 96-well ELISA plates were coated overnight at 4 °C with Wuhan-strain SARS-CoV-2 receptor-binding domain (RBD; 1 *μ*g per well) diluted in carbonate coating buffer (0.05 M sodium carbonate, pH 9.6). Plates were washed with PBST (PBS supplemented with 0.05% (v/v) Tween-20) and blocked with 5% (w/v) non-fat dry milk in PBS for 1 h at room temperature. Samples were applied as five serial dilutions and incubated for 1 h at room temperature with gentle shaking. After washing with PBST to remove unbound material, bound VHHs were detected using an HRP-conjugated anti-His antibody (Ay63, Cat. HIS-PLM535). Each dilution was assayed in triplicate. An anti-Huntingtin antibody (clone EM48, Cat. MAB5374) was included as a negative control. Controls included periplasmic extract from *E. coli* BL21 cells harboring empty pET-22b(+) vector (negative control) and VHH C5 (7OAO), a characterized anti-RBD nanobody (positive control). Signal was developed using the Bio-Rad Opti 4-CN substrate kit (Cat. 1708235) according to the manufacturer’s instructions, and absorbance was measured on a microplate reader. Protein expression was confirmed by dot blot using an HRP-conjugated anti-His antibody (Ay63, Cat. HIS-PLM535). A purified His-tagged recombinant protein was included as a positive control for anti-His detection, whereas an unrelated His-tagged protein and extracts from *E. coli* BL21 cultured without plasmid were included as negative controls for antigen specificity/background. Western blot analysis was performed using the same anti-His antibody, with extracts from BL21 cells without plasmid serving as the negative control.

### Localized surface plasmon resonance

LSPR measurements were performed on a Nicoya OpenSPR-XT instrument using a High-Capacity Carboxyl sensor (Nicoya) and a PBS-T pH 7.4 running buffer (1× PBS, 0.05% Tween-20). Binding experiments were performed at room temperature. Analyte VHH samples were diluted to the indicated concentrations in PBS-T. RBD was used as the ligand immobilized to the carboxyl sensor and Ab-157 was used as the analyte in solution. Carboxyl sensor surfaces were activated with 10 mM 1-ethyl3-(3-dimethylaminopropyl) carbodiimide (EDC) and N-hydroxy succinimide (NHS) in ultrapure water (MilliQ, resistivity 18.2 MΩ·cm). RBD was prepared at 50 *μ*g/mL in sodium acetate pH 5.0 and coupled to the surface to ~1800 RU at a flow rate of 10 *μ*L/min. RBD immobilization gave a stable immobilization response with minimal surface degradation over time. Unreacted NHS esters were blocked with 1 M ethanolamine prior to analyte injection. Binding and unbinding experiments were run at a flow rate of 40 *μ*L/min. Between each measurement cycle, the sensor surface was regenerated with two 10-second injections of 10 mM Glycine-HCl pH 2.0 to remove bound Ab-157 from the RBD surface. A 1:1 binding model with simultaneous curve fitting was applied. The kinetic results were analyzed with the manufacturer suggested analysis in TraceDrawer 1.9.1. The analysis included subtraction of the reference channel from the immobilized active channel of RBD.

### Prediction of antibody structure and computational docking to the target antigen

Variable heavy-chain sequences identified by ASPred were folded using IgFold (57), and the resulting structures were refined with PyRosetta (81). Antibody sequences were renumbered according to the Chothia scheme (82). The RBD structure was generated with AlphaFold2 (83) and used as the ligand, whereas antibody structures were used as receptors in the ClusPro 2.0 docking web server (60, 84) with antibody mode and non-CDR masking enabled.

### Computational estimation of binding affinities

The Fast cOarse-grained pRotein-proTein modEl (FORTE) (62) is a coarse-grained biophysical model specifically designed to simulate protein-protein interactions, allowing for the dynamic adjustment of amino-acid charges on titratable groups based on the surrounding environment at a specified pH (input as a parameter). The core of this model is the FPTS (63) combined with the ability to translate and rotate macromolecules using the Metropolis Monte Carlo method. All calculations with FORTE were performed at pH 7.4, 150 mM NaCl, and 298K. For a more accurate incorporation of hydrophobic interactions, we used a Lennard-Jones potential energy with *ε*_*LJ*_ equivalent to 0.1736 kJ/mol. The heavy chains of the top candidates predicted by ASPred were folded using IgFold and then used as input for the FORTE simulations. The wildtype RBD structure was constructed via the SWISS-MODEL workspace, using the NCBI reference sequence NC_045512 (accession YP_009724390.1) as a template. The free energies of interaction (or binding affinities) were calculated as a function of the macromolecules’ separation distances by analyzing their center-to-center pair radial distribution functions. These values were sampled in histogram form during the MC production phase, providing detailed distributions of the probability of finding the two molecules at different separation distances. To compare binding affinities across systems, we adopted the free energy minima (*βA*) observed in these simulations, which represent the most stable interaction points. Following the equilibration phase, each system underwent at least 3 × 10^9^ MC steps for production-phase sampling. To account for variability, three independent replicates were conducted for each system, allowing for the estimation of statistical errors for a proper comparison between the simulated systems.

### Electrostatic stability of antibody candidates

The electrostatic stabilities of the antibody heavy chains were calculated directly from the averaged net charges obtained in the FPTS simulations, using the IgFold-generated conformations (85, 86). Coulombic contributions of individual titratable groups for each protein structure were measured. These stabilities were determined by evaluating the Coulombic contributions of individual titratable groups for each protein structure under specific conformation and defined physical–chemical conditions. This approach allows for an assessment of how electrostatic interactions may influence the stability of each antibody candidate.

### Molecular dynamics simulations

All-atom molecular dynamics simulations of antibody–RBD complexes were performed with GROMACS 2024.3 using the AMBER99SB-ILDN force field for proteins and the TIP3P water model. Each complex was placed in a cubic simulation box with a minimum solute–box-edge distance of 1.0 nm, solvated explicitly, and neutralized with counterions; NaCl was then added to a final concentration of 0.15 M. After energy minimization, each system was equilibrated for 100 ps in the NVT ensemble and 100 ps in the NPT ensemble at 300 K and 1 bar, respectively. Production simulations were then carried out for 300 ns with a 2 fs integration time step. Short-range Coulomb and van der Waals interactions were evaluated using a 1.0 nm cut-off, and long-range electrostatics were treated with the particle-mesh Ewald method. Temperature was controlled with the V-rescale thermostat (*τ* = 0.1 ps), applied separately to protein and solvent, and pressure was maintained with the Parrinello–Rahman barostat (*τ* = 2.0 ps; compressibility = 4.5 × 10 − ^5^ bar − ^1^). Bonds involving hydrogen atoms were constrained with the LINCS algorithm. Periodic boundary conditions were removed before downstream trajectory analyses. Trajectory stability was assessed using standard structural metrics, including backbone RMSD and residue-level fluctuations. Interfacial contacts, buried surface area, hydrogen bonds, salt bridges, and other residue–residue interaction features were analyzed using COCOMAPS (87) and are provided in Supplementary Data 13. Unless otherwise indicated, reported interface summaries correspond to the analyzed antibody–RBD complex structures derived from the simulation workflow described above.

### Statistical Methods

#### Determination of BEAM–ASPred overlap

We evaluated ASPred on heavy-chain V(D)J sequences from a human BEAM dataset in which B cells isolated from the blood of SARS-CoV-2-infected individuals were profiled for RBD-binding specificity. The dataset consisted of whole PBMCs processed using the Single Cell BEAM Ab Immune Profiling workflow (Cell Ranger v3.1.0; proprietary data provided by 10x Genomics). We quantified the overlap between sequences classified as positive by ASPred and sequences classified as positive by BEAM (Supplementary Data 11). The paired dataset also included linked scRNA-seq profiles, enabling differential gene expression analysis between antigen-specific (AS^+^) and non-specific (AS−) B cells using Seurat (v5.0.1) and the Wilcoxon rank-sum test. Cell identities were assigned with SingleR.

#### Diversity of BCRs and estimation of sequence similarity to the training data

To assess whether predicted antigen-specific BCRs (AS^+^) represented previously unobserved solutions or were closely related to sequences in the training set, we quantified their sequence-level novelty using a nearest-neighbor (NN) similarity framework based on normalized Levenshtein distances (88). Briefly, CDRH3 amino acid sequences from the AS^+^ subset were compared to all antigen-specific heavy-chain sequences used for model training. For each query sequence *q*_*i*_, the highest normalized Levenshtein similarity to any training sequence *t*_*j*_ was computed as:

$$s\left(q_{i},t_{j}\right)=1-\frac{d_{Lev}\left(q_{i},t_{j}\right)}{\text{max}\left(\left|q_{i}\right|,\left|t_{j}\right|\right)}$$

where *d*_*Lev*_(*q*_*i*_,*t*_*j*_) denotes the standard Levenshtein edit distance between the two sequences, and *s*(*q*_*i*_,*t*_*j*_) ∈ [0,1] corresponds to identical sequences when *s* = 1. The nearest-neighbor similarity for a query was defined as *S*_*i*_ = max_*j*_ *s*(*q*_*i*_,*t*_*j*_). Computations were performed using the RapidFuzz implementation for efficiency. When specified, comparisons were restricted to same-length CDR3s to approximate Hamming similarity. A novelty threshold *T* was defined empirically using a “negation” reference set comprising antigen-unrelated or AS^+^ BCRs. The nearest-neighbor similarities of these negation sequences to the training set were computed, and *T* was chosen as the (1 − *δ*)-quantile of their similarity distribution, corresponding to a false-positive tolerance *δ* = 0.01:

$$T=Q_{1-\delta }\left(S_{\text{negation}\to \text{train}}\right).$$

AS^+^ sequences with best NN similarity *S*_*i*_ < *T* were considered *novel*. Per-donor novelty frequencies were summarized as the fraction of AS^+^ BCRs classified as novel, with 95% Wilson confidence intervals (89).

#### Phylogenetic analysis of antigen-specific sequences

We identified the intersection between ASPred-positive and BEAM-positive sequences using a probability threshold of 0.5, yielding 127 AS^+^ sequences. AS− sequences from healthy human repertoires were used as antigen-nonspecific controls. We then generated 200 FASTA files, each containing the same 127 AS^+^ sequences together with 127 randomly sampled AS^−^ control sequences. Each multifasta file was aligned with MAFFT using the auto setting. Maximum-likelihood phylogenies were inferred with RAxML-NG (MPI) under the LG + G4 + F amino-acid substitution model, with 1000 standard nonparametric bootstrap replicates per dataset. For each tree, we computed Fitch parsimony for AS^+^/AS^−^ labels and compared the observed value with a permutation-based null distribution (*n* = 999). We also recorded the sizes of AS^+^only clades containing at least three descendants and quantified local AS^+^ enrichment as the mean fraction of AS^+^ sequences among the *k* = 3 nearest neighbors (See corresponding data and code in Supplementary Data 14).

#### Gradient-based saliency attribution and IMGT mapping

To interpret sequence features driving ASPred predictions, we computed per-token saliency scores using a gradient-based attribution approach. For each input sequence, we backpropagated the logit corresponding to the antigen-associated (binder) class through the fine-tuned ESM-2 classifier and quantified token importance as the L2 norm of the gradient of the logit with respect to the embedding output at each token position–gradient saliency (89). Padding positions were masked using the model attention mask.

Saliency was computed with respect to the embedding outputs of the ESM-2 backbone after Low-Rank Adaptation (LoRA) fine-tuning. A forward hook was registered on the embedding module to replace the embedding output with a cloned tensor requiring gradient tracking, ensuring that gradients with respect to individual token embeddings could be captured during backpropagation. For each sequence, gradients were aggregated across the embedding dimension to yield a single scalar importance score per token. Attribution analysis was applied to training sequences pooled across all antigen classes.

To enable alignment of saliency profiles across sequences of varying length, tokenlevel saliency scores were mapped to standardized IMGT positions using ANARCI with the IMGT numbering scheme. Only canonical amino acid tokens were retained, and special or padding tokens were excluded from downstream analyses. The resulting IMGT-mapped saliency tables were used for position-aligned analyses of distributed sequence features across framework and complementarity-determining regions.

## Supplementary Material

Supplementary Files

This is a list of supplementary files associated with this preprint. Click to download.

- PacoetalGenomeMedicineIntialsubSUPPLEMENT.docx

## Data Availability

All data are available in the main text or the supplementary materials (listed below with links). Each Supplementary Data item includes a README file describing file contents, column definitions, and how the files map to the corresponding analyses in the manuscript. All codes and associated model-related data are available from: https://github.com/karenpaco/ASPred Numerical values underlying all main-text figures are provided as Source Data. Additional processed data supporting the findings of this study are available in the Supplementary Data files or from the corresponding authors upon reasonable request.
